# Chemical Composition, Antioxidant Activity, Anti-Fatigue Function and Mechanism of Pomegranate Peel Polyphenols on Exercise-Induced Fatigue in Mice

**DOI:** 10.3390/foods15091576

**Published:** 2026-05-03

**Authors:** Xing-Yu Ma, Yu-Mei Wang, Yu-Dong Hu, Bin Wang, Li Xu

**Affiliations:** Zhejiang Provincial Engineering Technology Research Center of Marine Biomedical Products, School of Food and Pharmacy, Zhejiang Ocean University, Zhoushan 316022, China; maxingyu@zjou.edu.cn (X.-Y.M.); wangyumei@zjou.edu.cn (Y.-M.W.); 18768049480@163.com (Y.-D.H.)

**Keywords:** pomegranate peel polyphenols (PPPs), LC-MS/MS, antioxidant activity, anti-fatigue function, Keap1/Nrf2 pathway, AMPK/PGC-1α/PPAR-α pathway, molecular docking

## Abstract

Pomegranate peel is a food industry waste rich in polyphenols. To date, its effect in alleviating fatigue remains unclear. This study aimed to characterize the chemical composition of pomegranate peel polyphenols (PPPs), evaluate its antioxidant and anti-fatigue capacities, and investigate the underlying mechanism. In the current study, twenty main compounds, primarily flavonoids, phenolic acids, and anthocyanins, were identified from PPPs using LC-MS/MS. In H_2_O_2_-induced HepG2 cells, PPPs promoted cellular repair and reduced the production of intracellular malondialdehyde (MDA) and reactive oxygen species (ROS) via enhancing the activity of antioxidant enzymes (SOD, CAT, and GSH-Px). In the endurance swimming-induced fatigue mice model, PPPs prolonged mice exhaustion times, reduced accumulation of fatigue-related metabolites (BUN, LA, BA, LDH and CK), and alleviated liver and muscle tissue damage. Mechanistically, PPPs mitigated oxidative stress via activation of the Keap1/Nrf2 pathway, leading to increased expression of hemeoxygenase-1 (HO-1) and NAD(P)H quinone oxidoreductase 1 (NQO1). Furthermore, PPPs stimulated energy metabolism by activating the AMPK/PGC-1α/PPAR-α pathway, promoting mitochondrial biogenesis, enhancing glycogen storage, increasing ATPase activity (Na^+^-K^+^-ATPase, Ca^2+^-Mg^2+^-ATPase, and T-ATPase) and accelerating lipid β-oxidation. These findings suggest that PPPs is a promising anti-fatigue supplement and could be further utilized in the nutritional industry.

## 1. Introduction

Fatigue is a common health concern in modern society and is characterized by persistent tiredness, reduced physical performance, and diminished capacity to maintain routine activities [1,2]. Prolonged or persistent fatigue may disrupt immune homeostasis and increase the risk of multiple disorders, including chronic diseases, cancer, depression, and autoimmune diseases, thereby posing a serious threat to human health [3,4,5]. Although several pharmacological agents are clinically used for fatigue management, their long-term application is markedly limited by delayed onset of efficacy and unavoidable adverse side effects [6]. In contrast, natural food supplements are gaining increasing attention as viable therapeutic alternatives, primarily due to their safety profiles and validated efficacy [7]. Therefore, the investigation of natural anti-fatigue functional food supplements is urgently needed.

Exercise-induced fatigue is a complex physiological process, and several theories have been proposed to explain its underlying mechanisms. The clogging theory suggests that strenuous exercise promotes the buildup of fatigue-related metabolites, such as blood urea nitrogen (BUN) and lactic acid (LA), thereby impairing muscle function and accelerating fatigue onset [8]. The energy exhaustion theory proposes that strenuous or prolonged exercise causes excessive depletion of energy substrates, resulting in insufficient energy availability and ultimately leading to fatigue [9]. In addition, the oxidative stress theory posits that strenuous exercise disrupts the balance between endogenous oxidant and antioxidant systems, leading to excessive reactive oxygen species (ROS) production and oxidative damage in tissues, which further contributes to fatigue development. Among these, the oxidative stress theory has garnered significant attention [10]. Consequently, investigations centered around these theories have emerged as a focal point in the realm of anti-fatigue supplements.

Polyphenols, a diverse group of bioactive phytochemicals, are recognized for their potent antioxidant, anti-inflammatory, and immunomodulatory properties [11]. Notably, emerging research has particularly highlighted the anti-fatigue properties of polyphenols through multiple mechanistic pathways [12]. For instance, areca nut polyphenols exert anti-fatigue effects by modulating the gut–liver axis, leading to enhanced hepatic energy metabolism and restoration of redox balance [13]. Hawthorn polyphenol microcapsules have also been shown to alleviate fatigue by regulating Nrf2-mediated oxidative stress and the AMPK pathway in skeletal muscle [14]. In addition, *Lonicera caerulea* berry polyphenols mitigate fatigue by targeting oxidative stress, inflammation, and apoptosis in skeletal muscle [15]. Collectively, polyphenols are promising candidates for the development of natural anti-fatigue functional nutraceuticals.

Pomegranate (*Punica granatum* L.), a deciduous fruit-bearing shrub, is rich in polyphenolic compounds, including flavonoids, phenolic acids, anthocyanins, and hydrolyzable tannins, which contribute to its diverse biological activities [16]. During pomegranate processing, however, the peel is commonly discarded as an agro-industrial byproduct, despite accounting for a substantial proportion of the fruit mass and containing higher levels of polyphenols than the edible pulp or juice [17]. Increasing evidence indicates that the remarkable antioxidant, anti-inflammatory, antimicrobial, and hepatoprotective activities of pomegranate peel are closely associated with its rich polyphenolic constituents, highlighting the considerable potential of pomegranate peel polyphenols (PPPs) for value-added utilization [18,19]. Considering the potential involvement of oxidative stress in exercise-induced fatigue, PPPs may represent promising natural candidates for fatigue alleviation. Nevertheless, the anti-fatigue potential of PPPs has not yet been systematically investigated. In particular, limited information is available regarding its chemical composition, protective effects against oxidative injury, and efficacy in exercise-induced fatigue models. Therefore, the present study aimed to characterize the chemical composition of PPPs, methodically assess its antioxidant and anti-fatigue effects, and further explore the underlying mechanism by establishing the H_2_O_2_-induced oxidative injured cell model and the exhaustive swimming fatigue model of mice. This research will provide experimental evidence and a theoretical basis for the application of PPPs in fatigue reduction-related functional foods.

## 2. Materials and Methods

### 2.1. Materials and Reagents

Pomegranate peel polyphenols (PPPs) (purity > 90%) was obtained from Shanxi Jinrun Biotechnology Co., Ltd. (Xi’an, China). The total polyphenol content of PPPs was further verified in our laboratory via the Folin–Ciocalteu method with gallic acid as the reference standard, and consistent quality was confirmed in three independent batches.

Methanol, acetonitrile, formic acid, isopropanol, phosphate buffer saline (PBS), 1,1-diphenyl-2-pic-rylhydrazyl (DPPH) and 2,2′-azinobis-(3-ethylbenzthiazoline-6-sulphonate) (ABTS), iron sulfate (FeSO_4_), reduced disodium salt hydrate (NADH), and nitrotetrazolium blue chloride (NBT) were provided by Sigma-Aldrich Trading Co., Ltd. (Shanghai, China). Dulbecco’s modified eagle medium (DMEM), fetal bovine serum (FBS), RPMI-1640, MTT and DMSO were supplied by Beijing Solarbio Technology Co., Ltd. (Beijing, China). Assay kits of BUN, BG, BA, LD, LDH, CK, LG, MG, TNF-α, IL-β, and IL-6 were obtained from Nanjing Jiancheng Bioengineering Research Institute (Nanjing, China). Assay kits of Na^+^-K^+^-ATPase, Ca^2+^-Mg^2+^-ATPase and T-ATPase were purchased from Hangzhou Leyi Bio-technology Co., Ltd. (Hangzhou, China). HepG2 cells were sourced from the Cell Bank of Type Culture Collection of the Chinese Academy of Sciences (Shanghai, China). The antibodies of Nrf2, Keap1, NQO1, HO-1, Phospho-AMPK (p-AMPK), AMPK, TFAM, PPAR-α, CPT-1, and PGC1-α were obtained from Wuhan Sanying Bio-technology Co., Ltd. (Wuhan, China).

### 2.2. LC-MS/MS Analysis Method

LC-MS/MS analysis of PPPs was conducted by Shanghai Meiji Biomedical Technology Co., Ltd. (Shanghai, China). Samples were analyzed using a UHPLC-Q Exactive HF-X system (Thermo Fisher Scientific, Waltham, MA, USA) equipped with an ACQUITY UPLC HSS T3 column (100 mm × 2.1 mm, 1.8 μm; Waters, Milford, MA, USA) maintained at 40 °C. The mobile phase consisted of solvent A (95% water, 5% acetonitrile, 0.1% formic acid) and solvent B (47.5% acetonitrile, 47.5% isopropanol, 5% water, 0.1% formic acid), with a flow rate of 0.40 mL/min. In positive ion mode, the elution gradient was as follows: 0–3 min, 0–20% B; 3–4.5 min, 20–35% B; 4.5–5 min, 35–100% B; 5–6.3 min, 100% B; 6.3–6.4 min, 100–0% B; and 6.4–8 min, 0% B. In negative ion mode, the elution gradient was: 0–1.5 min, 0–5% B; 1.5–2 min, 5–10% B; 2–4.5 min, 10–30% B; 4.5–5 min, 30–100% B; 5–6.3 min, 100% B; 6.3–6.4 min, 100–0% B; and 6.4–8 min, 0% B. A constant injection volume of 3.0 μL was used.

Mass spectrometric detection was performed in both positive and negative ion modes. The system parameters were as follows: scan range of m/z 70–1050, sheath gas flow rate of 50 arb, auxiliary gas flow rate of 13 arb, heater temperature of 425 °C, capillary temperature of 325 °C, spray voltage of ±3500 V, S-lens RF level at 50, and normalized collision energies of 20, 40, and 60 eV. The full MS and MS^2^ resolutions were set at 60,000 and 7500, respectively. Raw spectral data were processed with Progenesis QI software (version 3.0) to perform peak alignment and extract features. The identification of compounds was conducted with precision using databases.

### 2.3. Antioxidant Assay

The antioxidant activities were evaluated through multiple experimental approaches as follows: (1) DPPH radical scavenging activity was determined following the method described by Meng et al. with minor modifications [20,21]; (2) hydroxyl radical scavenging activity was assessed using the protocol established by Zhang et al. [22]; (3) superoxide anion (·O_2_^−^) radical scavenging capacity was analyzed based on the procedure developed by Sheng et al. [23]; (4) lipid peroxidation inhibition was assessed using a commercial assay kit according to the manufacturer’s instructions; and (5) iron-reducing power was evaluated following the methodology reported by Arise et al. [24].

### 2.4. Cytoprotective Effect of PPPs on H_2_O_2_-Induced HepG2 Cell Model

#### 2.4.1. Cytotoxicity Assay and Establishment of H_2_O_2_-Induced Cell Model

HepG2 cells were cultured in DMEM supplemented with 10% FBS and 1% penicillin streptomycin at 37 °C under 5% CO_2_ in a humidified sterile environment. After 24 h of cultivation, the culture medium was replaced with fresh medium containing PPPs or vitamin C (VC) at final concentrations of 5, 10, 20, 40, 80, 160, and 320 μg/mL, with a final volume of 200 μL per well. The cells were further incubated for 4 h under the same conditions. Subsequently, 10 μL of CCK-8 solution was added to each well in the dark, followed by incubation for 1 h at 37 °C. The absorbance at 450 nm was measured using a microplate reader.

Cell viability (%) = (OD_sample_/OD_control_) × 100 (1)

The H_2_O_2_-induced oxidative injured HepG2 cell model was established using procedures previously described in the literature [25]. HepG2 cells were seeded in 96-well plates at a density of 1 × 10^4^ cells per well, with 200 µL of medium added to each well, and subsequently cultured for 24 h. Once the cell density reached approximately 80% confluence, the culture medium was replaced with fresh medium containing various final concentrations of H_2_O_2_ (100, 150, 200, 250, 300, 350, and 400 μM), with each well containing 200 µL of medium. Subsequently, cell viability was assessed as described above.

#### 2.4.2. Effect of PPPs on the Viability of H_2_O_2_-Induced HepG2 Cells

HepG2 cells were cultured for 24 h, then pretreated with PPPs at 10, 20, and 40 μg/mL for another 24 h. Afterward, the cells were exposed to 400 μM H_2_O_2_ for 4 h. VC (40 μg/mL) served as the positive control. Cell viability was measured using the CCK-8 method as outlined in Section 2.4.1.

#### 2.4.3. Determination of Intracellular ROS, MDA and Antioxidant Enzyme Activities

HepG2 cells were incubated with PPPs (10, 20, and 40 μg/mL) for 24 h and then challenged with 400 μM H_2_O_2_ for 4 h to trigger oxidative injury. After washing with PBS, the cells were stained with 10 μM DCFH_2_-DA for 1 h in the dark. Fluorescence images were taken with a TI-S inverted fluorescence biomicroscope, and quantitative analysis was performed using ImageJ software (version 1.52v). Meanwhile, the levels of MDA content and the activities of SOD, CAT, and GSH-Px were detected using commercial kits in strict accordance with the manufacturer’s protocols.

### 2.5. Evaluation of PPPs in an Exercise-Induced Fatigue Mouse Model

#### 2.5.1. Animal Breeding and Experimental Design

**Figure 1:**
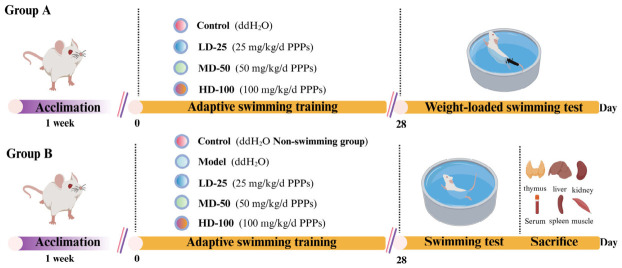
Experimental scheme for evaluating the anti-fatigue effects of PPPs in mice (*n* = 10).

Ninety 6-week-old male ICR mice (22 ± 2 g) were obtained from Hangzhou Ziyuan Laboratory Animal Science and Technology Co., Ltd. (Hangzhou, China, Certificate No. SYXK2019-0031). The mice were housed under controlled conditions (23 ± 2 °C, 45 ± 5% relative humidity, 12 h/12 h light/dark cycle). The experimental protocol is outlined in Figure 1. After one week of acclimatization, 90 male ICR mice were randomly divided into two main experimental groups to investigate the anti-fatigue effect of PPPs.

Group A was subjected to the weight-loaded exhaustive swimming test (detailed in Section 2.5.2) for exercise endurance evaluation. This group included four subgroups (*n* = 10 per subgroup): control, low-dose PPPs, medium-dose PPPs, and high-dose PPPs. All mice were administered by daily gavage for 28 consecutive days: the control group received 0.2 mL distilled water, while the low-, medium-, and high-dose groups were given PPPs at 25, 50, and 100 mg/kg bw/day, respectively.

Group B was assigned to serum and tissue sample collection following a 30 min swimming test, and consisted of five subgroups (*n* = 10 per subgroup): control (0.2 mL distilled water, no swimming), model (0.2 mL distilled water, swimming), and three PPPs-treated groups (25, 50, 100 mg/kg bw/day, swimming). Each mouse served as an independent biological replicate.

#### 2.5.2. Establishment of the Fatigue Model of Endurance Swimming Mice

According to the method described [26], 30 min after the final gavage, mice in group A were subjected to the exhaustive swimming test. The mice were placed individually in a specially designed swimming pool with a water depth of 30 cm and a constant temperature of 25 °C. A lead weight equivalent to 5% of each mouse’s body weight was attached to the tail. During the test, the water was continuously circulated to prevent the mice from floating or resting. Swimming time was recorded from the beginning of the test until exhaustion, which was defined as the inability of the mouse to rise to the water surface for breathing within 8 s after submersion. The time to exhaustion was recorded as the exhaustive swimming time.

#### 2.5.3. Measurement of Body Weight and Organ Index of Mice

Throughout the experimental period, daily body weight measurements were systematically recorded for all mice, and all the mice received standard diet mouse food daily and had access to water freely. Upon completion of the intervention, blood samples were collected from the mice’s eyeballs in group B. Additionally, the liver, leg muscles, kidneys, spleen, and thymus were carefully dissected. Individual organs were weighed using a precision analytical balance to determine organ indices. The organ index was calculated using the following Formula (2):

Organ index (%) = (Weight of organ/Weight of mouse) × 100(2)

#### 2.5.4. Histopathological Analysis

The liver and muscle tissues were immersed in 4% paraformaldehyde and fixed overnight. Subsequently, the tissues were embedded in paraffin, and sections approximately 4 µm thick were prepared. These sections underwent staining with hematoxylin and eosin (H&E). Micrographs were captured using a light microscope.

#### 2.5.5. Detection of Biochemical Indicators

Following 28 days of intervention, ICR mice in group B underwent retro-orbital blood collection 30 min post-swimming cessation, and the supernatant was centrifuged at 4000 rpm for 10 min and stored for further use. After humane euthanasia via cervical dislocation, liver and muscle tissues of the mice were excised, weighed using precision scales, and homogenized in ice-cold 0.9% NaCl (1:9 w/v) with a mechanical homogenizer. The resulting 10% homogenates were centrifuged (4000 rpm, 20 min) to obtain supernatant fractions. The levels of LA, BUN, BG, BA, LDH, and CK in the serum were measured using commercially available assay kits. The levels of LG, MG, MDA, SOD, GSH-Px, Na^+^-K^+^-ATPase, Ca^2+^-Mg^2+^-ATPase, T-ATPase, IL-1β, IL-6, and TNF-α in the liver and muscle tissue were quantified using specific assay kits, according to the instructions of the corresponding kit.

#### 2.5.6. Western Blot Analysis

Protein extracts were obtained from mouse liver tissue using RIPA lysis buffer supplemented with protease and phosphatase inhibitors. The Western blotting procedure was carried out following the reported method [27]. The protein concentration was quantified using the bicinchoninic acid (BCA) assay. Following extraction, proteins were separated by electrophoresis on 12% SDS-polyacrylamide gels and then electrotransferred onto PVDF membranes. The membranes were sequentially incubated with primary and secondary antibodies, then detected through enhanced chemiluminescence (ECL). Protein band visualization and quantification were performed using FluorChem FC3 imaging software (AlphaView version 6.0).

### 2.6. Molecular Docking Analysis

Molecular docking experiments were performed as previously described [20]. The receptor proteins Keap1 (PDB ID: 2FLU) and AMPK (PDB ID: 4CFF) were obtained from the RCSB protein database (https://www.rcsb.org/, accessed on 23 March 2026) [28,29]. The 3D compound structures, including quercetin, caffeic acid, epicatechin, and gallic acid were obtained from PubChem Download (https://pubchem.ncbi.nlm.nih.gov/, accessed on 23 March 2026). Affinities were determined through docking analysis using AutoDock Vina (version 1.1.2) and Pymol (version 2.4.0) with default configurations, and the results were visualized in 2D and 3D plots using Discovery Studio (version 2019).

### 2.7. Statistical Analysis

Experimental data were expressed as mean ± standard deviation (SD). In vitro experiments were performed with three independent biological replicates (*n* = 3), and in vivo animal experiments included 10 mice per subgroup (*n* = 10). Prior to statistical analysis, data normality and variance homogeneity were assessed. One-way analysis of variance (ANOVA) was employed to compare differences among all experimental groups (control, model and treatment groups), followed by Tukey’s test to identify significant differences (*p* < 0.05).

## 3. Results

### 3.1. Chemical Analysis of PPPs by LC-MS/MS

**Figure 2:**
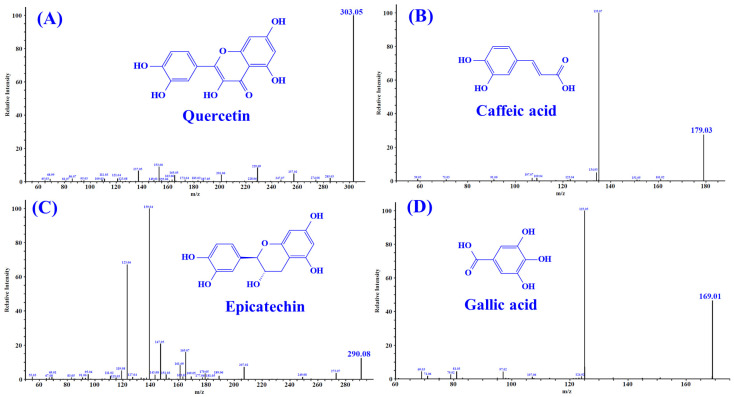
Secondary mass spectra of four important substances identified from pomegranate peel polyphenols (PPPs): quercetin (**A**); caffeic acid (**B**); epicatechin (**C**); gallic acid (**D**).

Untargeted metabolomic profiling of PPPs was performed through dual-polarity LC-MS/MS analysis with the characteristic fragmentation patterns illustrated in Figure S1. The chemical composition of PPPs is summarized in Table 1. A total of 20 major components were identified, mainly including flavonoids, phenolic acids, and anthocyanins. Previous studies have demonstrated that these compounds displayed significant antioxidant and anti-fatigue functions [30,31,32,33]. In addition, according to the literature, quercetin [34], caffeic acid [35], epicatechin [36], and gallic acid [37] may be four key bioactive compounds in PPPs. These constituents are likely to play crucial roles in mediating the biological activities of PPPs, particularly its antioxidative and anti-fatigue properties. The MS/MS spectra of quercetin (m/z 303.05, formula C_15_H_10_O_7_), caffeic acid (m/z 179.03, formula C_9_H_8_O_4_), epicatechin (m/z 290.08, formula C_15_H_14_O_6_), and gallic acid (m/z 169.01, formula C_7_H_6_O_5_) are presented in Figure 2.

### 3.2. Antioxidant Activity of PPPs In Vitro

**Figure 3:**
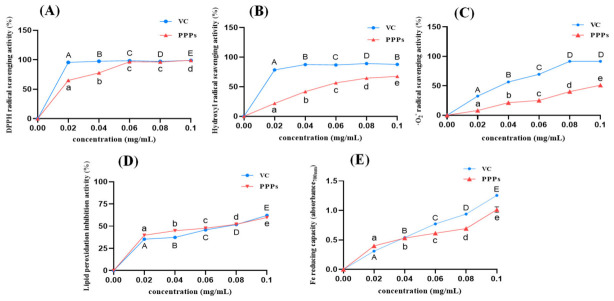
Antioxidant activity of PPPs (0.02–0.1 mg/mL) in vitro. DPPH radical scavenging activity (**A**); hydroxyl radical scavenging activity (**B**); ·O_2_^−^ scavenging activity (**C**); lipid peroxidation inhibition activity (**D**); and iron-reducing capacity (**E**). Ascorbic acid (VC) was used as a positive control. ^A–E^ or ^a–e^ Different letters indicate significant differences between groups (*p* < 0.05).

As shown in Figure 3A–E, PPPs exhibited considerable antioxidant activity in vitro, including DPPH, OH radical, and ·O_2_^−^ scavenging abilities, as well as inhibition of lipid peroxidation and iron-reducing capacity. Additionally, the antioxidant activity of PPPs at concentrations ranging from 0.02 to 0.10 mg/mL exhibited a continuous, dose-dependent enhancement. Notably, at 0.06 mg/mL, the DPPH radical scavenging activity and lipid peroxidation inhibitory effect of PPPs were already comparable to those of the positive control, indicating that PPPs exerted potent in vitro antioxidant activity.

### 3.3. Mitigating Function of PPPs on H_2_O_2_-Induced HepG2 Cell Model

#### 3.3.1. PPPs Protected Against H_2_O_2_-Induced Damage in HepG2 Cells

**Figure 4:**
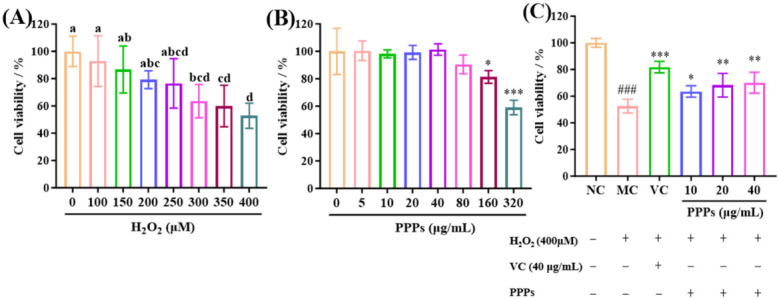
PPPs protected against H_2_O_2_-induced damage in HepG2 cells. Effect of H_2_O_2_ (0–400 μM) on the viability of HepG2 cells (**A**); effect of PPPs on the viability of HepG2 cells (**B**); effect of PPPs on the viability of H_2_O_2_-induced HepG2 cells (**C**). ^a–d^ Different letters indicate significant differences between groups (*p* < 0.05); ^###^ *p* < 0.001 vs. control group (NC), *** *p* < 0.001, ** *p* < 0.01, * *p* < 0.05 vs. model group (MC).

Cell viability, a direct measure of cellular damage, is used to establish the oxidative stress model. As shown in Figure 4A, H_2_O_2_ treatment decreased HepG2 cell viability in a concentration-dependent manner, and 400 μM H_2_O_2_ reduced viability to approximately 50% of the control value. Therefore, this concentration was selected to establish the oxidative injury model for subsequent experiments [38]. As illustrated in Figure 4B, PPPs showed no significant cytotoxicity toward HepG2 cells at concentrations of 5–80 μg/mL compared with the control group (*p* > 0.05). Therefore, 10, 20, and 40 μg/mL were selected as the low-, medium-, and high-dose PPPs groups, respectively [39]. As shown in Figure 4C, VC and PPPs significantly increased the viability of H_2_O_2_-treated HepG2 cells compared with the model group (*p* < 0.05), indicating that PPPs protected HepG2 cells against H_2_O_2_-induced oxidative injury.

#### 3.3.2. Effects of PPPs on Oxidative Stress in Cells

**Figure 5:**
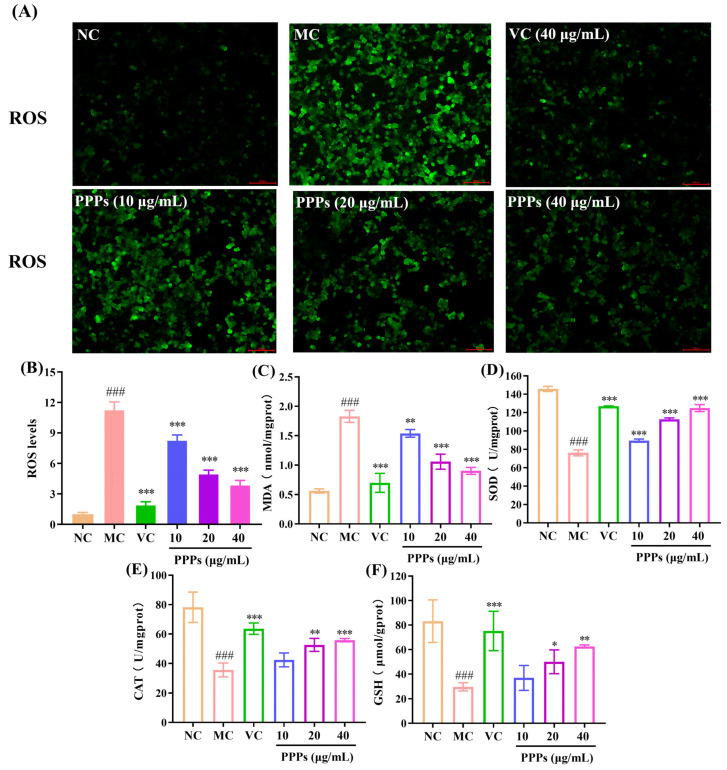
Effects of PPPs on H_2_O_2_-induced oxidative stress in HepG2 cells. ROS production (**A**) and analysis (**B**) in HepG2 cells; the levels of MDA (**C**), SOD (**D**), CAT (**E**), and GSH-Px (**F**) in HepG2 cells. NC: normal control; MC: model group; VC: positive control; PPPs (10 μg/mL): low-dose group of PPPs; PPPs (20 μg/mL): medium-dose group of PPPs; PPPs (40 μg/mL): high-dose group of PPPs. ^###^ *p* < 0.001 vs. NC; * *p* < 0.05, ** *p* < 0.01 and *** *p* < 0.001 vs. MC.

The changes in intracellular ROS fluorescence are presented in Figure 5A. The model group demonstrated the highest ROS production, pointing to cell damage compared to the control group. Treatment with PPPs decreased the fluorescence intensity effectively. Figure 5B illustrated the effect of PPPs on intracellular ROS levels in HepG2 cells, as measured by relative fluorescence intensity. H_2_O_2_ induction led to a significant increase in ROS levels (*p* < 0.001) compared to the control. Treatment with PPPs effectively attenuated ROS generation in a concentration-dependent manner, with all tested concentrations (10, 20, and 40 μg/mL) significantly reducing ROS levels compared to the model group (*p* < 0.001). Notably, 40 μg/mL PPPs decreased the relative fluorescence intensity by 65.43%, indicating a strong inhibitory effect on H_2_O_2_-induced ROS overproduction.

As illustrated in Figure 5C–F, H_2_O_2_ treatment markedly disrupted the antioxidant defense system in HepG2 cells. Compared with the control group, the activities of SOD, CAT, and GSH-Px were significantly decreased, whereas the MDA content was significantly increased (*p* < 0.001). However, PPPs treatment effectively reversed these changes. In particular, after treatment with 40 μg/mL PPPs, the activities of SOD, CAT, and GSH-Px were significantly elevated (*p* < 0.01), while the MDA level was markedly reduced (*p* < 0.001) compared with the model group. Results indicated that PPPs is an effective antioxidant that could significantly improve the oxidative damage of HepG2 cells induced by H_2_O_2_.

### 3.4. Effect of PPPs on Exercise-Induced Fatigue Model in Mice

#### 3.4.1. Effect of PPPs on Body Weight and Organ Index of Fatigue Model of Mice

As shown in Table 2, after 28 days of gavage, all groups of mice exhibited an increase in body weight. However, no statistically significant differences in body weight were observed among the control, model, and PPPs-treated groups, indicating that the weight gain was part of normal growth. Additionally, the organ indices of the liver, kidney, spleen, and thymus gland in the low-, medium-, and high-dose PPPs groups did not differ significantly from those of the model group (*p* > 0.05). These findings indicated that gavage administration of PPPs combined with 30 min forced swimming did not adversely affect the growth or development of the mice, which were maintained under standard housing and feeding conditions.

#### 3.4.2. Effect of PPPs on Tissue Morphology of Fatigue Model of Mice

**Figure 6:**
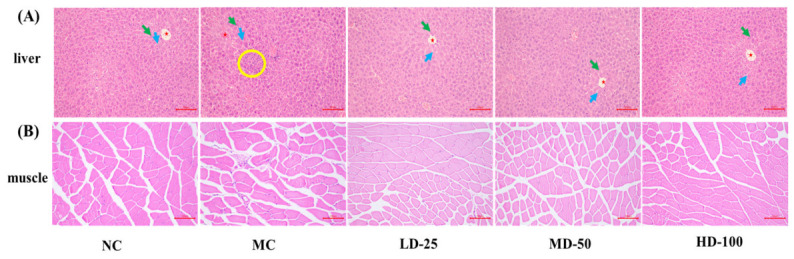
H&E staining of mice tissue samples (100×, scale bar = 100 μm). Mouse liver tissue samples (**A**) and muscle tissue samples (**B**). The fatigue model of mice was established by an endurance swimming method. NC: normal control; MC: model group; LD-25: low-dose group of PPPs (25 mg/kg·bw/d); MD-50: medium-dose group of PPPs (50 mg/kg·bw/d); HD-100: high-dose group of PPPs (100 mg/kg·bw/d). The blue arrow indicates the hepatic sinusoids, the green arrow indicates the hepatic cord, the red star shows the central vein, and the yellow circle shows inflammatory cells.

As illustrated in Figure 6A, liver nuclei in the control group remained intact, with hepatocytes displaying no necrosis in H&E staining. Hepatic cords (green arrows) and sinusoids (blue arrows) were radially arranged around the central vein (red star), with clear structures and no inflammatory cell or macrophage infiltration. In contrast, the model group exhibited disordered hepatic cords, enlarged and unclear sinusoids, and mild inflammatory cell infiltration (yellow circles) near the central vein. PPPs treatment significantly restored liver architecture, improving cord arrangement, clarifying sinusoids, and preventing local necrosis and macrophage infiltration. Notably, 100 mg/kg PPPs nearly normalized liver tissue, with no significant difference from controls. Overall, PPPs administration effectively alleviated liver oxidative stress after 30 min of fatigue swimming. Figure 6B demonstrated that gastrocnemius muscle fibers in the control group were uniformly aligned with distinct transverse striations, whereas the model group showed irregular fiber arrangement and muscle damage. Treatment with PPPs dose-dependently reduced muscle fiber damage, demonstrating the protective effect of PPPs against muscle injury in mice.

#### 3.4.3. Determination of Exhaustive Swimming Time

**Figure 7:**
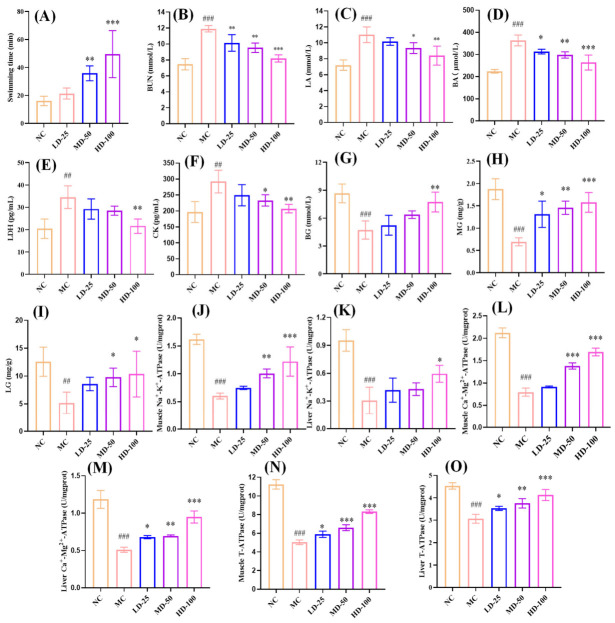
Effect of PPPs on exercise capacity and biochemical indices related to fatigue in mice. Exercise capacity (**A**); BUN (**B**); LA (**C**); BA (**D**); LDH (**E**); CK (**F**); BG (**G**); MG (**H**); LG (**I**); muscle Na^+^-K^+^-ATPase (**J**); liver Na^+^-K^+^-ATPase (**K**); muscle Ca^2+^-Mg^2+^-ATPase (**L**); liver Ca^2+^-Mg^2+^-ATPase (**M**); muscle T-ATPase (**N**); liver T-ATPase (**O**). ^###^ *p* < 0.001, ^##^ *p* < 0.01 compared to control group. *** *p* < 0.001, ** *p* < 0.01 and * *p* < 0.05 compared to model group.

Exercise tolerance assay is the most direct and objective method for assessing physical fatigue [40]. In this study, the anti-fatigue effects of PPPs were evaluated using the weight-loaded swimming test, in which time to exhaustion was recorded as an indicator of exercise tolerance and fatigue severity. As shown in Figure 7A, the control group showed a time to exhaustion of 16.00 ± 3.34 min. In contrast, all PPPs-administered groups demonstrated significantly prolonged exhaustion times, reaching 21.34 ± 3.92 min (low dose), 35.80 ± 5.30 min (medium dose), and 49.55 ± 16.80 min (high dose). In particular, there was a significant difference in the medium- and high-dose PPPs groups compared with the model group (*p* < 0.01). Furthermore, as the dose of PPPs increased, the exhaustive swimming time of the mice was progressively prolonged, indicating that PPPs exerted anti-fatigue effects in a dose-dependent manner.

#### 3.4.4. Effect of PPPs on Serum Biomarkers Related to Fatigue

To investigate the metabolic changes associated with the anti-fatigue activity of PPPs, key serum biochemical parameters were measured in mice, including markers of energy metabolism (lactic acid, LA), byproducts of protein catabolism (blood urea nitrogen, BUN), indicators of nitrogen metabolism (blood ammonia, BA), and enzymes associated with tissue damage (lactate dehydrogenase and creatine kinase, LDH and CK) [41]. As shown in Figure 7B–F, following endurance swimming exercise in mice, the levels of BUN, LA, BA, LDH and CK increased notably compared to the control group (*p* < 0.01). However, PPPs intervention effectively reversed the changes in these biomarkers. Particularly in the high-dose PPPs group, the levels of BUN, LA, BA, LDH and CK were significantly decreased by 31.21%, 23.79%, 27.44%, 37.62% and 29.09%, respectively, compared with the model group (*p* < 0.01). These findings suggested that PPPs alleviated fatigue and muscle damage caused by strenuous exercise via regulating energy metabolism and exercise homeostasis.

#### 3.4.5. Effect of PPPs on BG, MG, and LG of Fatigue Model of Mice

Maintaining stable blood sugar (BG) is essential for the proper functioning of the body. During exercise, glycogen, particularly muscle glycogen (MG) and liver glycogen (LG), serves as a major energy reserve, and its depletion is closely associated with fatigue [42]. As shown in Figure 7G–I, the fatigued model mice exhibited significantly lower levels of BG, MG, and LG compared with the control group (*p* < 0.01), confirming successful fatigue modeling. PPPs administration dose-dependently restored these energy-related indicators. In particular, 100 mg/kg PPPs significantly increased BG, MG, and LG levels by 63.77%, 128.99%, and 100.39%, respectively, compared with the model group (*p* < 0.05). These findings indicated that PPPs enhanced blood glucose availability, promoted glycogen storage, and modulated energy metabolism, thereby improving exercise tolerance in fatigued mice.

#### 3.4.6. Effect of PPPs on Na^+^-K^+^-ATPase, Ca^2+^-Mg^2+^-ATPase, and T-ATPase Activities of Fatigue Model of Mice

Na^+^-K^+^-ATPase, Ca^2+^-Mg^2+^-ATPase, and T-ATPase serve as essential enzymatic regulators in cellular energy metabolism through ATP generation [9]. As shown in Figure 7J–O, endurance swimming markedly suppressed Na^+^-K^+^-ATPase, Ca^2+^-Mg^2+^-ATPase, and T-ATPase activities in both muscle and liver tissues of the model group compared to controls (*p* < 0.001). PPPs administration restored these enzyme activities in a dose-dependent manner. Specifically, 100 mg/kg PPPs significantly increased Na^+^-K^+^-ATPase activity by 62.67% in muscle (*p* < 0.001) and 90.32% in liver (*p* < 0.05), compared with the model group. Ca^2+^-Mg^2+^-ATPase activity was also increased by 111.25% in muscle and 84.31% in liver after high-dose PPPs treatment (*p* < 0.001). In addition, T-ATPase activity was elevated by 65.41% in muscle and 34.64% in liver in the 100 mg/kg PPPs group, compared with the model group (*p* < 0.001). These findings indicated that PPPs effectively mitigated exercise-induced reductions in key ATPase activities in muscle and liver.

#### 3.4.7. Effect of PPPs on Antioxidant and Anti-Inflammatory Capacities in Fatigue Model of Mice

**Figure 8:**
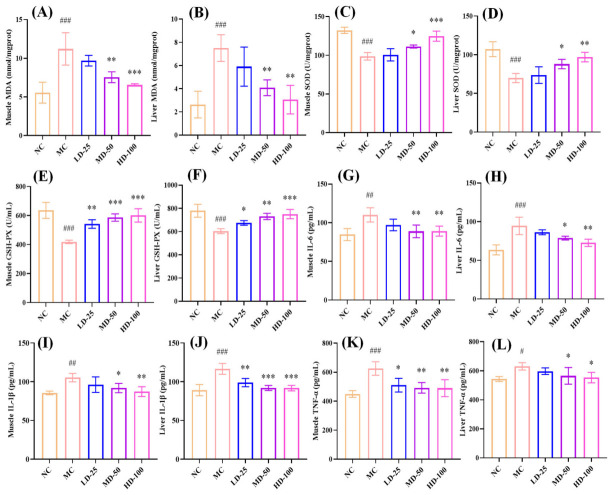
Effect of PPPs on anti-inflammatory and antioxidant capacity in fatigue model mice. Muscle MDA (**A**); liver MDA (**B**); muscle SOD (**C**); liver SOD (**D**); muscle GSH-Px (**E**); liver GSH-Px (**F**); muscle IL-6 (**G**); liver IL-6 (**H**); muscle IL-1β (**I**); liver IL-1β (**J**); muscle TNF-α (**K**); liver TNF-α (**L**). ^###^ *p* < 0.001, ^##^ *p* < 0.01, ^#^ *p* < 0.05 compared to control group. *** *p* < 0.001, ** *p* < 0.01 and * *p* < 0.05 compared to model group.

As illustrated in Figure 8A–F, exhaustive swimming markedly decreased the activities of SOD and GSH-Px and elevated MDA levels in both muscle and liver tissues (*p* < 0.001). However, after treatment with PPPs, the above phenomena were significantly reversed. Following administration of 100 mg/kg PPPs, the activities of SOD and GSH-Px enzymes in both muscle and liver tissues returned close to normal levels, coincident with a significant reduction in MDA content (*p* < 0.01). These results indicated that PPPs has the potential to diminish liver and muscle injury by alleviating oxidative stress.

Inflammatory cytokines and ROS engage in a bidirectional relationship, where ROS induce cytokine production via signaling pathways, and cytokines further enhance ROS generation, promoting inflammation and oxidative stress-mediated tissue damage [43,44]. As shown in Figure 8G–L, exhaustive swimming significantly up-regulated the expression levels of IL-6, IL-1β, and TNF-α in liver and muscle of mice (*p* < 0.05), which was improved after PPPs administration. These findings indicated that PPPs mitigated oxidative stress-induced damage and fatigue by lowering inflammatory factor levels.

#### 3.4.8. Effect of PPPs on Protein Expression of the Keap1/Nrf2 Signaling Pathway in Fatigue Model of Mice

**Figure 9:**
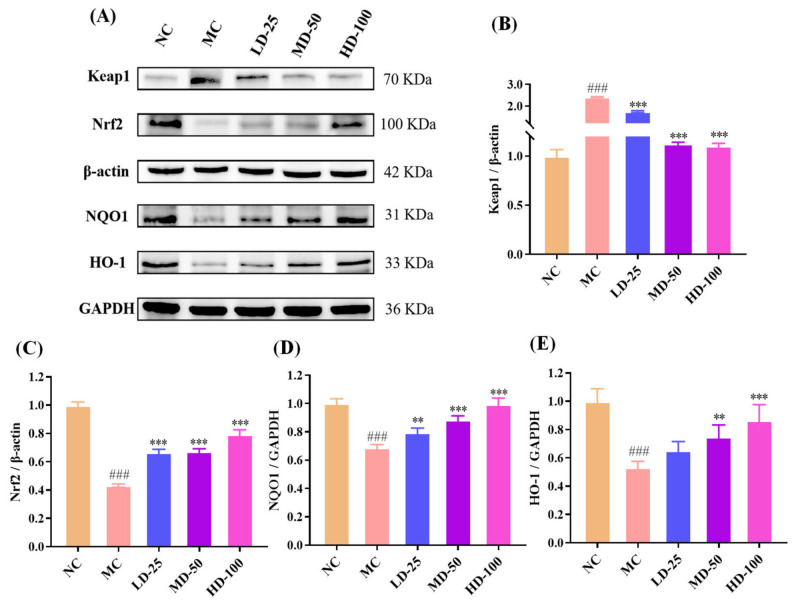
Effect of PPPs on Keap1/Nrf2 signaling pathway in fatigue model mice. Western blot images (**A**). Effect of PPPs on expression levels of Keap1 (**B**), Nrf2 (**C**), NQO1 (**D**), and HO-1 (**E**) protein in mice. ^###^ *p* < 0.001 compared to control group. *** *p* < 0.001 and ** *p* < 0.01 compared to model group.

The Keap1/Nrf2 pathway is a critical protective signaling axis against oxidative stress [45]. As shown in Figure 9A–E, exhaustive swimming markedly increased Keap1 protein expression while significantly decreasing the expression levels of Nrf2, NQO1, and HO-1 in the model group compared with the control group (*p* < 0.001), indicating suppression of the antioxidant defense system in fatigued mice. PPPs administration markedly reversed these changes, as evidenced by the down-regulation of Keap1 and the up-regulation of Nrf2 and its downstream antioxidant proteins, NQO1 and HO-1. These results suggested that PPPs may mitigate oxidative stress-related damage and fatigue through modulation of the Keap1/Nrf2 signaling pathway and enhancement of the endogenous antioxidant system.

#### 3.4.9. Effect of PPPs on Protein Expression of the AMPK/PGC1-α/PPAR-α Signaling Pathway in Fatigue Model of Mice

**Figure 10:**
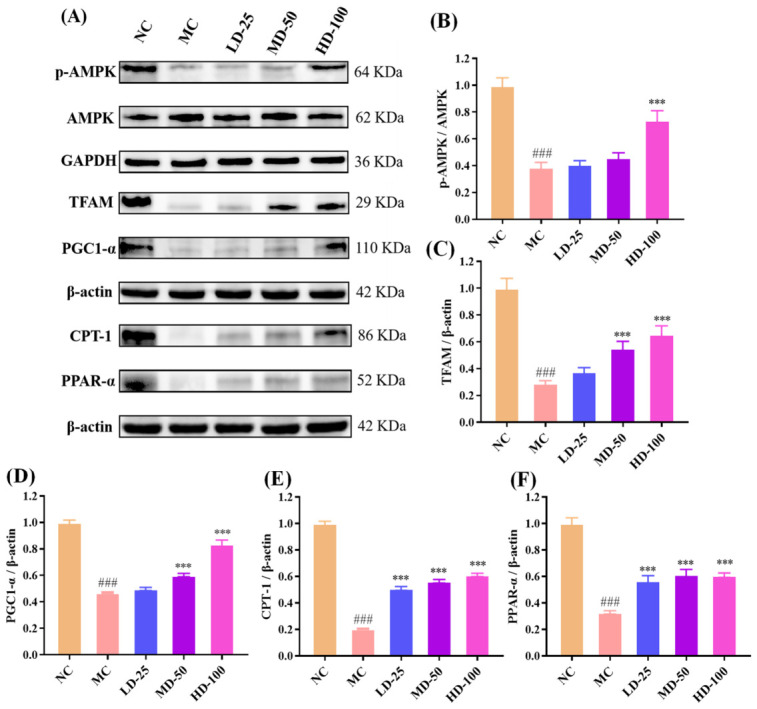
Effect of PPPs on AMPK/PGC1-α/PPAR-α signaling pathway in fatigue model mice. Western blot images (**A**). Effect of PPPs on expression levels of p-AMPK (**B**), TFAM (**C**), PGC1-α (**D**), CPT-1 (**E**), and PPAR-α (**F**) protein in mice. ^###^ *p* < 0.001 compared to control group. *** *p* < 0.001 compared to model group.

Mitochondrial bioenergetic dysfunction constitutes a key pathological basis for exercise-induced fatigue. The AMPK/PGC-1α/PPAR-α signaling pathway plays a critical regulatory role in maintaining energy homeostasis [46,47]. As shown in Figure 10A–E, exhaustive swimming significantly down-regulated the protein expression levels of p-AMPK, PGC-1α, TFAM, CPT-1, and PPAR-α in the model group compared with the control group (*p* < 0.001), suggesting suppression of pathways associated with mitochondrial biogenesis and fatty acid oxidation. PPPs administration significantly reversed these alterations, as evidenced by increased phosphorylated AMPK and up-regulated expression of PGC-1α, TFAM, CPT-1, and PPAR-α. These findings suggested that PPPs may alleviate fatigue by activating the AMPK/PGC-1α/PPAR-α signaling pathway, thereby promoting mitochondrial biogenesis, enhancing fatty acid β-oxidation, and improving energy homeostasis.

### 3.5. Molecular Docking Results

#### 3.5.1. Molecular Docking Results of Quercetin, Caffeic Acid, Epicatechin, and Gallic Acid with Keap1 Protein

To further explore the potential mechanism by which PPPs regulate the Keap1/Nrf2 pathway, molecular docking was performed between Keap1 and four major bioactive components identified in PPPs, including quercetin, caffeic acid, epicatechin, and gallic acid. The Kelch domain of Keap1 contains multiple ligand-binding pockets (P1–P5), which are formed by residues involved in hydrogen bonding and hydrophobic interactions and are closely associated with ligand recognition [20,28].

**Figure 11:**
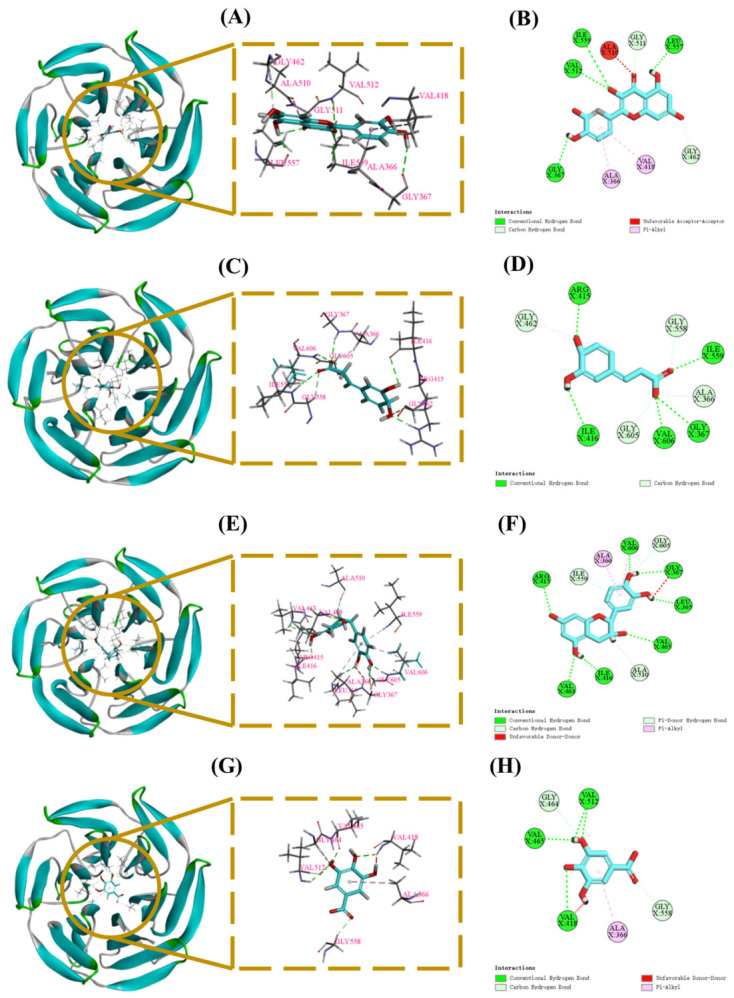
Molecular docking results of quercetin, caffeic acid, epicatechin, and gallic acid. 3D map of the interaction between Keap1 protein and quercetin (**A**), caffeic acid (**C**), epicatechin (**E**), and gallic acid (**G**), respectively. 2D plot of the interaction between Keap1 protein and quercetin (**B**), caffeic acid (**D**), epicatechin (**F**), and gallic acid (**H**), respectively.

As shown in Figure 11A–H and Table 3, four bioactive components of PPPs exhibited favorable binding to the Kelch domain of Keap1. The predicted binding energies of quercetin, caffeic acid, epicatechin, and gallic acid were −9.9, −7.1, −8.9, and −6.7 kcal/mol, respectively, suggesting favorable binding affinities. Among them, quercetin, caffeic acid, and epicatechin were predicted to interact with key residues Gly462 and/or Arg415 located in the P1 pocket, suggesting a binding mode associated with the canonical ligand recognition region of Keap1. In contrast, gallic acid showed a distinct interaction pattern, forming hydrogen bonds with Val465, Gly464, Val512, Gly558, and Val418, together with a hydrophobic interaction with Ala366. Overall, these docking results provide in silico support for the notion that PPPs may contribute to the activation of the Keap1/Nrf2 pathway by targeting the Keap1 Kelch domain.

#### 3.5.2. Molecular Docking Results of Quercetin, Caffeic Acid, Epicatechin, and Gallic Acid with AMPK Protein

**Figure 12:**
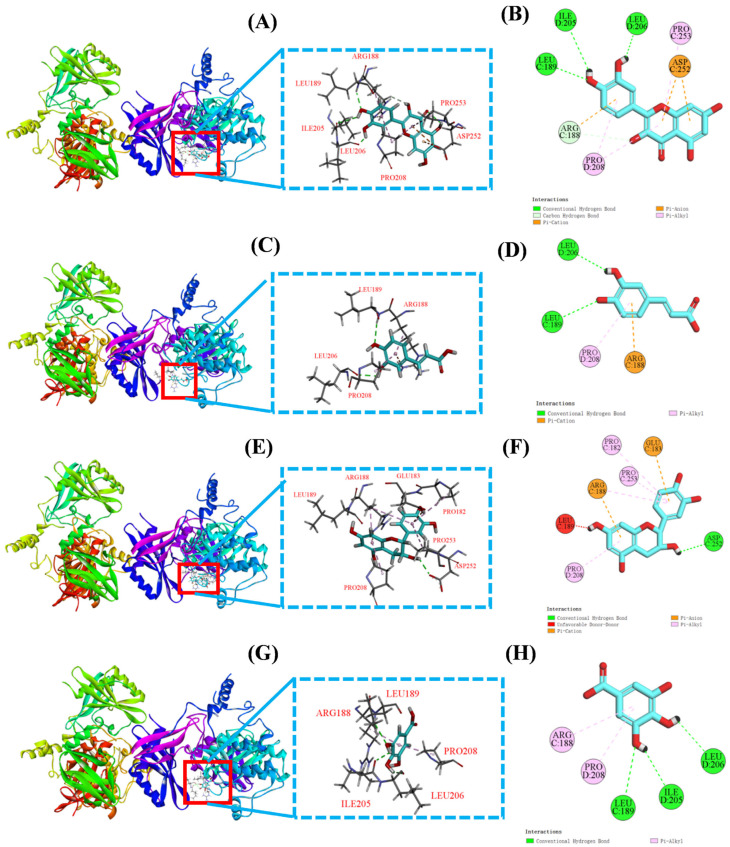
Molecular docking results of AMPK protein with quercetin, caffeic acid, epicatechin, and gallic acid, respectively. 3D map of the interaction between AMPK protein and quercetin (**A**), caffeic acid (**C**), epicatechin (**E**), and gallic acid (**G**), respectively. 2D plot of the interaction between AMPK protein and quercetin (**B**), caffeic acid (**D**), epicatechin (**F**), and gallic acid (**H**), respectively.

Based on the finding that PPPs enhanced AMPK phosphorylation and up-regulated the expression of PGC-1α, TFAM, CPT-1, and PPAR-α in fatigue model mice (Figure 10), molecular docking was performed to further explore the potential interactions between AMPK and four major bioactive components identified in PPPs, including quercetin, caffeic acid, epicatechin, and gallic acid. AMPK is a heterotrimeric complex consisting of a catalytic α subunit and regulatory β and γ subunits. Given the critical role of the α subunit in AMPK signaling, AMPKα was selected for docking analysis [29]. As shown in Figure 12A–H and Table 4, all four bioactive components exhibited favorable binding to AMPKα. The predicted binding energies of quercetin, caffeic acid, epicatechin, and gallic acid were −7.6, −5.7, −7.1, and −5.5 kcal/mol, respectively, suggesting favorable binding affinities. Consequently, these docking results provided in silico support for the potential involvement of PPPs in the modulation of the AMPK signaling pathway through interaction with AMPKα.

## 4. Discussion

**Figure 13:**
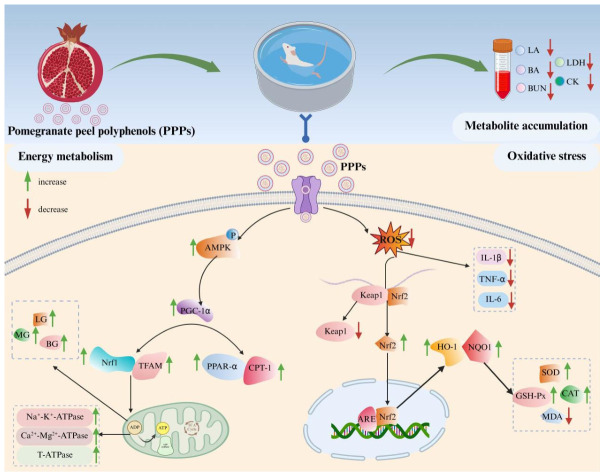
The mechanism of PPPs in alleviating fatigue in mice.

Fatigue, a multifactorial physiological condition characterized by a transient decline in physical performance and functional capacity, has become an increasingly prevalent health concern in modern society [3,4,5]. However, currently available clinical interventions for fatigue management remain limited, lacking clear mechanisms and effective functional food supplements [48]. Therefore, there is a pressing need to develop novel food-derived bioactive compounds with robust anti-fatigue properties. Among these, bioactive phytochemicals derived from dietary sources present a promising avenue due to their multimodal mechanisms of action targeting mitochondrial biogenesis, oxidative stress modulation, and energy metabolism regulation [49]. Consequently, this study was designed to investigate the chemical composition of PPPs, assess its antioxidant and anti-fatigue effects, and elucidate the underlying mechanism by an H_2_O_2_-induced oxidative damage cell model and endurance swimming fatigue model in mice. The results showed that PPPs exhibited significant antioxidant and anti-fatigue properties, with quercetin, caffeic acid, epicatechin, and gallic acid being potentially key contributors to these effects. Collectively, the anti-fatigue effects of PPPs appeared to involve three main aspects: (1) reducing the accumulation of fatigue-related metabolic byproducts, (2) enhancing hepatic glycogen storage, and (3) alleviating oxidative stress through the maintenance of redox homeostasis (Figure 13) [8,9,10].

PPPs are naturally occurring low-molecular-weight compounds comprising flavonoids, phenolic acids, and anthocyanins. These polyphenols have been extensively studied for their diverse physiological activities, notably their antioxidant and anti-fatigue potential. Previous studies have demonstrated that quercetin can improve fatigue resistance by mitigating oxidative damage, promoting glycogen storage, and enhancing muscle endurance [34]. Similarly, caffeic acid has been shown to alleviate chronic fatigue through attenuation of oxidative damage, regulation of mitochondrial enzyme complex activities, and optimization of mitochondrial redox homeostasis [35]. Notably, epicatechin significantly enhances exercise tolerance by activating the PGC-1α signaling pathway, thereby promoting mitochondrial biogenesis and augmenting skeletal muscle oxidative capacity [36]. Furthermore, gallic acid mitigates fatigue by enhancing mitochondrial function, modulating energy metabolism, and remodeling gut microbiota [37]. Therefore, we deduced that quercetin, caffeic acid, epicatechin, and gallic acid might be key bioactive constituents responsible for the antioxidative and anti-fatigue functions of PPPs.

Exercise tolerance is a primary indicator of anti-fatigue capacity. The exhaustive swimming test is widely considered a robust approach for assessing exercise tolerance due to its avoidance of mechanical damage, enforced experimental protocol, and generation of dependable data [40]. In the present study, PPPs significantly prolonged exhaustive swimming time in mice in a dose-dependent manner, suggesting a dose-dependent improvement in exercise tolerance. Histopathological analyses further demonstrated that PPPs attenuated fatigue-associated injury in liver and muscle tissues. These results collectively supported the protective effect of PPPs against exercise-induced fatigue.

According to the metabolite accumulation theory, fatigue during intense exercise is closely related to the excessive generation and insufficient clearance of metabolic byproducts (such as LA, BUN, BA, LDH and CK) [8]. During vigorous exercise, muscles obtain sufficient energy through anaerobic glycolysis, leading to LA accumulation.This is accompanied by a decrease in pH in muscles and blood, disrupting biochemical processes, affecting muscle contraction, and harming organs, thereby causing fatigue [28]. In the present study, PPPs administration reduced serum LA levels, suggesting that PPPs may alleviate fatigue by attenuating lactate accumulation. Prolonged physical exertion also enhances protein and amino acid catabolism, resulting in increased ammonia production and subsequent elevation of BA and BUN, which are commonly regarded as biochemical indicators of fatigue [50]. The levels of BUN and BA in PPPs-administered groups were markedly decreased in the present study, indicating that PPPs decreased protein catabolism for energy, enhanced adaptive capacity to physical load, and ultimately improved fatigue tolerance. In addition, elevated LDH and CK levels usually reflect muscle membrane damage and exercise-induced fatigue [51,52]. PPPs supplementation significantly lowered LDH and CK levels, further indicating that PPPs enhanced physical endurance by decreasing the accumulation of metabolic byproducts and alleviating physical fatigue (Figure 13).

The oxidative stress theory proposes that intense exercise accelerates the accumulation of ROS, which can interact with proteins, nucleic acids, and membrane lipids, causing oxidative stress and fatigue. The antioxidant defense system against ROS mainly comprises enzyme systems, such as SOD, CAT, and GSH-Px [41]. In vitro, PPPs markedly enhanced SOD, CAT, and GSH-Px activities and reduced ROS and MDA levels in H_2_O_2_-treated HepG2 cells, indicating a protective effect against oxidative injury. Consistent with the in vitro observations, administration of PPPs significantly increased SOD and GSH-Px activities and markedly decreased MDA levels in both liver and muscle tissues of fatigued mice. Furthermore, inflammatory factors increase ROS production by activating intracellular signaling pathways and inducing ROS-producing enzymes, while ROS also act as signaling molecules that enhance inflammation, creating a positive feedback loop [44]. In the current study, PPPs treatment also significantly reduced the levels of inflammatory markers (IL-6, IL-1β, and TNF-α) in the liver and muscle of fatigued mice (Figure 13). These findings suggested that PPPs might mitigate liver and muscle injury and fatigue by improving redox balance and suppressing inflammatory responses. However, it should be noted that although HepG2 cells are widely used for oxidative stress screening, they do not recapitulate skeletal muscle physiology, which is central to exercise-induced fatigue. Therefore, future studies using physiologically relevant skeletal muscle models, such as C2C12 myotubes or primary skeletal muscle cells, combined with mechanistic interventions, are required to establish a direct link between the antioxidant effect of PPPs and its anti-fatigue efficacy.

The Keap1/Nrf2 signaling pathway serves as a master regulatory pathway orchestrating cellular antioxidant defense mechanisms [28]. Under physiological conditions, Nrf2 remains sequestered in the cytoplasm through binding with its repressor Keap1. Oxidative stress triggers Nrf2 liberation from Keap1-mediated degradation, enabling its nuclear translocation and subsequent binding to antioxidant response element (ARE) sequences in phase II detoxification enzyme gene promoters [53]. This molecular interaction initiates transcriptional activation of downstream cytoprotective genes, including NQO1 and HO-1, which collectively enhance cellular antioxidant capacity through coordinated regulation of redox homeostasis and detoxification pathways. In this study, PPPs significantly down-regulated the protein expression levels of Keap1 while up-regulating Nrf2, NQO1, and HO-1, indicating that PPPs effectively promoted Nrf2 nuclear translocation, activated downstream antioxidant enzymes, and consequently reduced ROS production to alleviate fatigue (Figure 13). Nevertheless, although PPPs treatment modulated oxidative stress-related markers and the expression of antioxidant signaling proteins, whether its anti-fatigue effect is directly mediated through the Keap1/Nrf2 pathway remains to be established. Future studies employing pathway inhibition or genetic loss-of-function strategies are warranted to clarify the precise contribution of Keap1/Nrf2 signaling to the anti-fatigue activity of PPPs.

The energy exhaustion theory emphasizes that extended exercise reduces glycogen stores in the muscles and liver (LG and MG), which consequently reduces blood glucose (BG) and induces fatigue. Increasing glycogen storage or minimizing glycogen consumption can enhance exercise endurance and postpone fatigue onset [54]. In the present study, PPPs supplementation significantly increased BG, LG, and MG levels, positively enhancing blood glucose utilization and promoting glycogen storage. In addition, ATPases including Na^+^-K^+^-ATPase, Ca^2+^-Mg^2+^-ATPase, and total T-ATPase are essential for material transport, energy transformation, and information transmission in physiological processes. Reduced ATPase activity may impair mitochondrial function and aggravate fatigue [55]. PPPs administration markedly increased the activities of these enzymes in liver and muscle tissues, indicating that PPPs may help maintain energy metabolism and ionic homeostasis under fatigue conditions, thereby reducing fatigue (Figure 13).

Mitochondrial function is closely related to fatigue resistance because mitochondria are central to ATP production and substrate utilization. The AMPK/PGC-1α/PPAR-α signaling network plays an important role in energy homeostasis, mitochondrial biogenesis, and fatty acid oxidation [56,57]. AMPK acts as a cellular energy sensor and activates PGC-1α through phosphorylation and deacetylation. Activated PGC-1α then promotes mitochondrial biogenesis by up-regulating downstream factors such as NRF and TFAM, which coordinate mitochondrial DNA replication and transcription to maintain energy homeostasis [50]. In parallel, PGC-1α coactivates PPARα, a key regulator of fatty acid uptake and β-oxidation, leading to increased CPT-1 expression and enhanced fatty acid catabolism, thereby improving energy supply for skeletal muscle during exercise [46]. In this study, we observed a significant increase in the protein expression of p-AMPK, PGC-1α, TFAM, PPAR-α, and CPT-1 in the PPPs-treated groups, suggesting that PPPs can enhance mitochondrial biosynthesis and fatty acid metabolism by regulating the AMPK/PGC-1α/PPAR-α signaling pathway, thereby promoting exercise tolerance and alleviating fatigue. Similarly, further studies using AMPK inhibition, gene silencing, or tissue-specific mechanistic approaches are needed to further elucidate the specific role of the AMPK/PGC-1α/PPAR-α signaling axis in mediating the anti-fatigue effects of PPPs.

Molecular docking was performed to explore potential interactions between key components of PPPs (quercetin, caffeic acid, epicatechin, and gallic acid) and two target proteins, Keap1 and AMPK. The docking results revealed that all four compounds exhibited favorable predicted binding affinities with Keap1. Additionally, quercetin, caffeic acid, and epicatechin were predicted to bind within the P1 pocket of the Keap1 Kelch domain, a key region mediating Nrf2 recognition. In contrast, gallic acid appeared to interact with Keap1 in a different manner, implying a possible non-competitive mode of action. Similarly, all four compounds also displayed favorable binding affinities with AMPKα in the docking analysis. These results suggest that PPPs may interact with key targets involved in the Keap1/Nrf2 and AMPK signaling pathways, thereby potentially contributing to reduced oxidative stress, improved metabolic homeostasis, and ultimately fatigue alleviation. These docking results provide preliminary mechanistic insights into the potential target interactions of PPPs. Nevertheless, further experimental investigations are warranted to validate these predicted interactions, confirm direct target engagement, and elucidate the precise molecular mechanism through which PPPs modulate the Keap1/Nrf2 and AMPK signaling pathways.

Overall, the present study provides evidence that PPPs exerts beneficial anti-fatigue effects, possibly through the coordinated regulation of oxidative stress and energy metabolism. However, as PPPs is a commercially available polyphenol preparation, future work would benefit from further efforts toward its standardization. In addition, clarifying the bioavailability, metabolic fate, tissue distribution, and in vivo active forms of PPPs will deepen the understanding of its functional basis. Further studies are also warranted to determine human-equivalent doses and to assess long-term safety and efficacy, thereby supporting the future development of PPPs as an anti-fatigue supplement in the nutritional industry.

## 5. Conclusions

In conclusion, this study systematically characterized the chemical composition of PPPs, assessed its antioxidant and anti-fatigue activities, and explored the potential underlying mechanism. Our findings demonstrated that PPPs exhibited potent antioxidant and anti-fatigue properties, significantly promoting cellular repair in H_2_O_2_-induced oxidative injury and effectively alleviating exercise-induced fatigue in mice. Mechanistically, these beneficial effects may be associated with the modulation of the Keap1/Nrf2 pathway, thereby alleviating oxidative damage, as well as the activation of the AMPK/PGC-1α/PPAR-α signaling axis, which may contribute to improved energy metabolism and related biochemical parameters. These findings provided a new approach for the further utilization, development, and industrial application of pomegranate peel processing byproducts. Future studies may focus on further clarifying the specific contributions of these signaling pathways to the anti-fatigue activity of PPPs and elucidating the underlying molecular mechanism in greater depth.

## Figures and Tables

**Table 1:** Chemical analysis of pomegranate peel polyphenols (PPPs) by LC-MS/MS.

Compound	*m*/*z*	Retention Time (min)	Formula	Fragmentation Score	CAS ID
gallic acid	169.01	1.69	C_7_H_6_O_5_	90.5	149-91-7
adenosine	268.10	1.90	C_10_H_13_N_5_O_4_	95.4	58-61-7
epicatechin	290.08	2.61	C_15_H_14_O_6_	83.4	490-46-0
caffeic acid	179.03	2.86	C_9_H_8_O_4_	80.2	331-39-5
proanthocyanins B_2_	577.14	2.97	C_30_H_26_O_12_	92.4	29106-49-8
4-coumaric acid	165.05	3.04	C_9_H_8_O_3_	85.9	4501-31-9
riboflavin	377.15	3.06	C_17_H_20_N_4_O_6_	91.7	83-88-5
protocatechuic acid	153.02	3.19	C_7_H_6_O_4_	91.2	99-50-3
ferulic acid	193.05	3.41	C_10_H_10_O_4_	87.2	537-98-4
isoquercetin	465.10	3.57	C_21_H_20_O_12_	92.9	482-35-9
genistein	271.06	3.79	C_15_H_10_O_5_	80.2	529-59-9
kaempferol	285.04	3.81	C_15_H_10_O_6_	63.8	520-18-3
hyperin	465.10	4.09	C_21_H_20_O_12_	95.4	482-36-0
quercetin	303.05	4.10	C_15_H_10_O_7_	88.6	117-39-5
kaempferol-3-O- rutinoside	595.17	4.37	C_27_H_30_O_15_	94.3	17650-84-9
isorhamnetin	317.07	4.48	C_16_H_12_O_7_	87.9	480-19-3
isoquercitrin	463.09	4.67	C_21_H_20_O_12_	67.9	482-35-9
astragalin	447.09	5.05	C_21_H_20_O_11_	81.6	480-10-4
biochanin A	285.08	5.28	C_16_H_12_O_5_	77.1	491-80-5

**Table 2:** Body weight and organ indices in mice (*n* = 10).

Characteristics		NC	MC	LD-25	MD-50	HD-100
Body Weight (g)	Initial	22.94 ± 1.01	23.10 ± 1.36	22.88 ± 0.79	22.51 ± 0.70	22.64 ± 0.53
	Final	36.80 ± 1.35	35.81 ± 2.91	35.66 ± 2.09	36.28 ± 1.49	35.76 ± 1.58
	Change	13.86 ± 0.34	12.71 ± 1.55	12.78 ± 1.30	13.77 ± 0.79	13.12 ± 1.05
Organ Index (%)	Liver	5.60 ± 0.30	5.68 ± 0.19	5.78 ± 0.26	5.68 ± 0.33	5.77 ± 0.31
	Kidney	1.65 ± 0.12	1.68 ± 0.13	1.66 ± 0.07	1.61 ± 0.08	1.68 ± 0.07
	Spleen	0.32 ± 0.03	0.30 ± 0.02	0.33 ± 0.02	0.32 ± 0.04	0.32 ± 0.03
	Thymus Gland	0.16 ± 0.04	0.15 ± 0.02	0.16 ± 0.03	0.15 ± 0.02	0.16 ± 0.02

All data are presented as the mean ± SD (*n* = 10).

**Table 3:** Molecular docking studies of quercetin, caffeic acid, epicatechin, and gallic acid in complex with Keap1 protein and their binding energies.

Binding Ligand	Amino Acid Residue That Interacts	Docking Score
quercetin	Hydrogen bonding: Gly367, Val512, Ile559, Gly511, Leu557, Gly462 Electrostatic forces: Ala366 and Val418	−9.9 kcal/mol
caffeic acid	Hydrogen Bonding: Gly462, Arg415, Gly558, Ile559, Ala366, Gly367, Val606, Gly605, Ile416	−7.1 kcal/mol
epicatechin	Hydrogen bonding: Arg415, Ile559, Val606, Gly605, Gly367, Leu365, Val465, Ala510, Ile416, Val463 Electrostatic forces: Ala366	−8.9 kcal/mol
gallic acid	Hydrogen bonding: Val465, Gly464, Val512, Gly558, Val418 Electrostatic forces: Ala366	−6.7 kcal/mol

**Table 4:** Molecular docking studies of quercetin, caffeic acid, epicatechin, and gallic acid in complex with AMPK protein and their binding energies.

Binding Ligand	Amino Acid Residue That Interacts	Docking Score
quercetin	Hydrogen bonding: Leu189, Ile205, Leu206, Arg188 Electrostatic interaction: Pro208, Pro253 Hydrophobic force: Asp252	−7.6 kcal/mol
caffeic acid	Hydrogen bonding: Leu206, Leu189 Electrostatic interaction: Pro208 Hydrophobic force: Arg188	−5.7 kcal/mol
epicatechin	Hydrogen bonding: Asp252 Electrostatic interaction: Pro182, Pro253, Pro208 Hydrophobic force: Glu183, Arg188	−7.1 kcal/mol
gallic acid	Hydrogen bonding: Leu206, Ile205, Ley189 Electrostatic interaction: Arg 188, Pro208	−5.5 kcal/mol

## Data Availability

The original contributions presented in this study are included in the article/Supplementary Materials. Further inquiries can be directed to the corresponding authors.

## Supplementary Materials

The following supporting information can be downloaded at: https://www.mdpi.com/article/10.3390/foods15091576/s1, Figure S1: Total ion chromatogram of pomegranate peel polyphenols (PPPs) by LC-MS/MS in positive (A) and negative ion modes (B), with the horizontal coordinate indicating the retention time of the peaks and the vertical coordinate indicating the relative total ion intensity. Figure S2: Raw Western blots depicting the impact of PPPs on Keap1/Nrf2 pathway protein expression (Keap1, Nrf2, NQO1, HO-1) in fatigue model mice. Figure S3: Raw Western blots depicting the impact of PPPs on AMPK/PGC1-α/PPAR-α pathway protein expression in fatigue model mice.

## Abbreviations

The following abbreviations are used in this manuscript:

ABTS	2,2′-azinobis-(3-ethylbenzthiazoline-6-sulphonate)
ANOVA	analysis of variance
AMPK	AMP-activated protein kinase
ARE	antioxidant response element
BA	blood ammonia
BCA	bicinchoninic acid
BG	blood glucose
BUN	blood urea nitrogen
CAT	catalase
CCK8	cell counting kit-8
CK	creatine kinase
CPT-1	carnitine palmitoyltransferase-1
DMSO	dimethyl sulfoxide
DMEM	dulbecco’s modified eagle medium
DPPH	1,1-diphenyl-2-pic-rylhydrazyl
GAPDH	glyceraldehyde 3-phosphate dehydrogenase
GSH-Px	glutathione peroxidase
H&E	hematoxylin-eosin staining
HepG2 cells	human hepatocellular carcinoma cell line
HO-1	heme oxygenase-1
IL-1β	interleukin-1β
IL-6	interleukin-6
Keap1	kelch-like ECH-associated protein l
LC-MS/MS	liquid chromatography tandem mass spectrometry
LA	lactic acid
LDH	lactate dehydrogenase
LG	liver glycogen
MDA	malondialdehyde
MG	muscle glycogen
NQO1	NAD(P)H dehydrogenase quinone 1
NBT	nitrotetrazolium blue chloride
Nrf2	nuclear factor erythroid 2-related factor 2
p-AMPK	phosphorylated AMP-activated protein kinase
PBS	phosphate buffer saline
PGC1-α	peroxisome proliferators-activated receptor γ coactivator 1α
PPARα	peroxisome proliferator-activated receptor-α
PPPs	pomegranate peel polyphenols
PVDF	polyvinylidene difluoride
SDS	sodium dodecyl sulfate
ROS	reactive oxygen species
SOD	superoxide dismutase
TFAM	recombinant transcription factor A, mitochondrial
TNF-α	tumor necrosis factor-α
VC	vitamin C
