# Supplementary material for: Chemical Composition, Antioxidant Activity, Anti-Fatigue Function and Mechanism of Pomegranate Peel Polyphenols on Exercise-Induced Fatigue in Mice
Source: Foods. 2026 May 3;15(9):1576. doi: 10.3390/foods15091576 (PMC13163272; doi:10.3390/foods15091576)
Supplement: Supplementary file 1 [file foods-15-01576-s001.zip › foods-4246274-supplementary.pdf]

Supplementary Material

Chemical Composition, Antioxidant Activity, Anti-Fatigue Function and Mechanism of Pomegranate Peel Polyphenols on Exercise-Induced Fatigue in Mice

Xing-Yu Ma, Yu-Mei Wang, Yu-Dong Hu, Bin Wang *, Li Xu *

Zhejiang Provincial Engineering Technology Research Center of Marine Biomedical Products, School of Food and Pharmacy, Zhejiang Ocean University, Zhoushan 316022, China.

***Corresponding Authors:**

Dr. Bin Wang, Tel./Fax: +86-580-2554818; E-mail: wangbin@zjou.edu.cn

Dr. Li Xu, Tel./Fax: +86 580-2554818; E-mail: 2022196@zjou.edu.cn

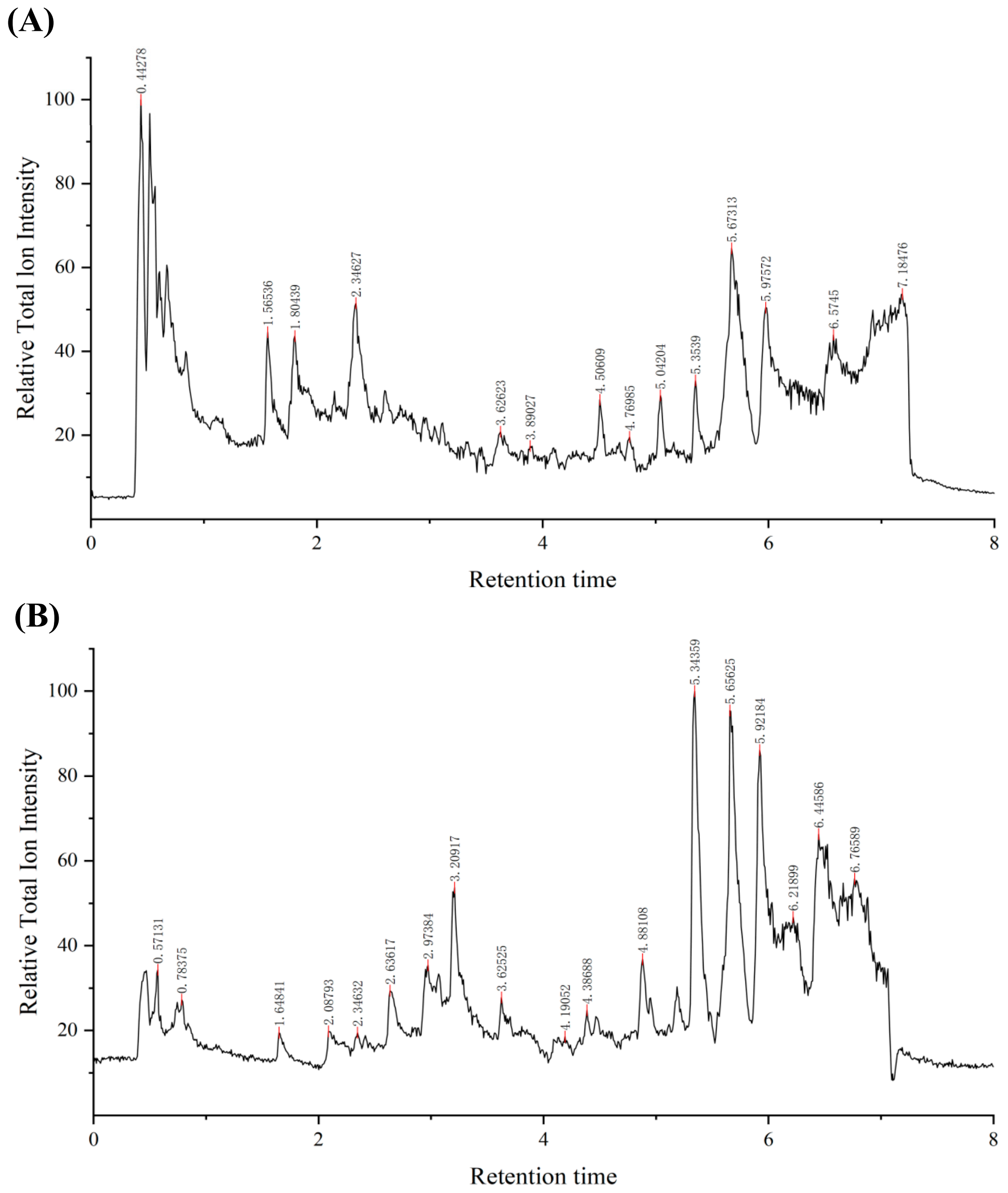

Fig. S1. Total ion chromatogram of pomegranate peel polyphenols (PPPs) by LC-MS/MS. Positive (A) and negative ion modes (B), with the horizontal coordinate indicating the retention time of the peaks and the vertical coordinate indicating the relative total ion intensity.

Fig. 9A-Keap1

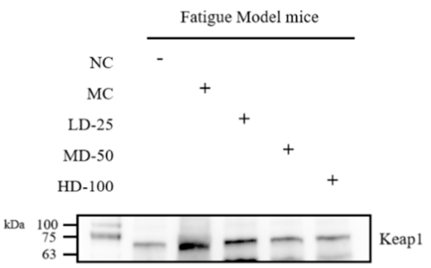

Fig. S2-Keap1

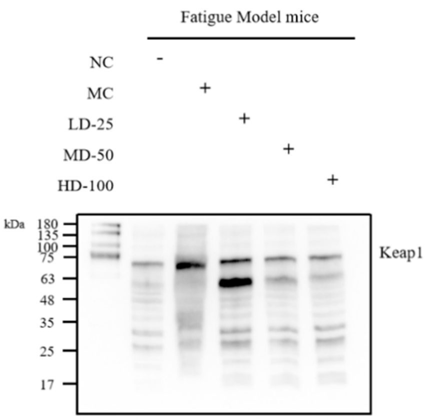

Fig. 9A-Nrf2

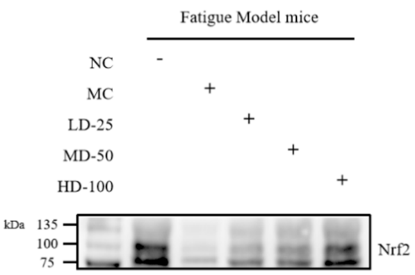

Fig. S2-Nrf2

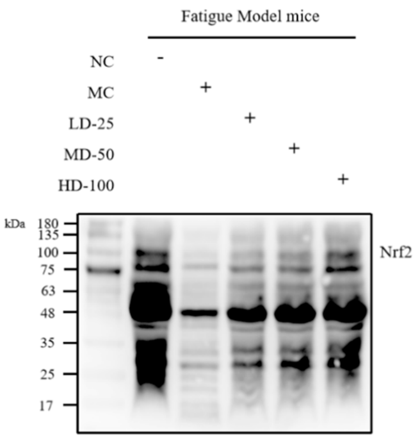

Fig. 9A-β-actin

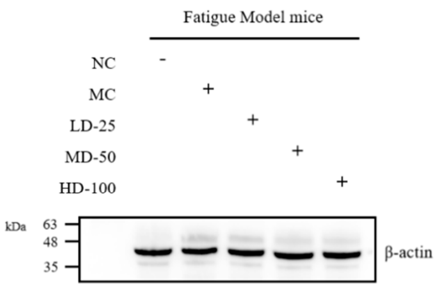

Fig. S2-β-actin

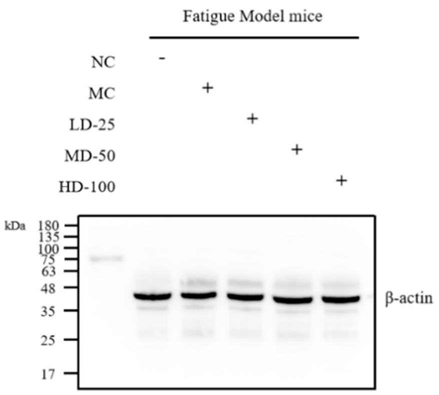

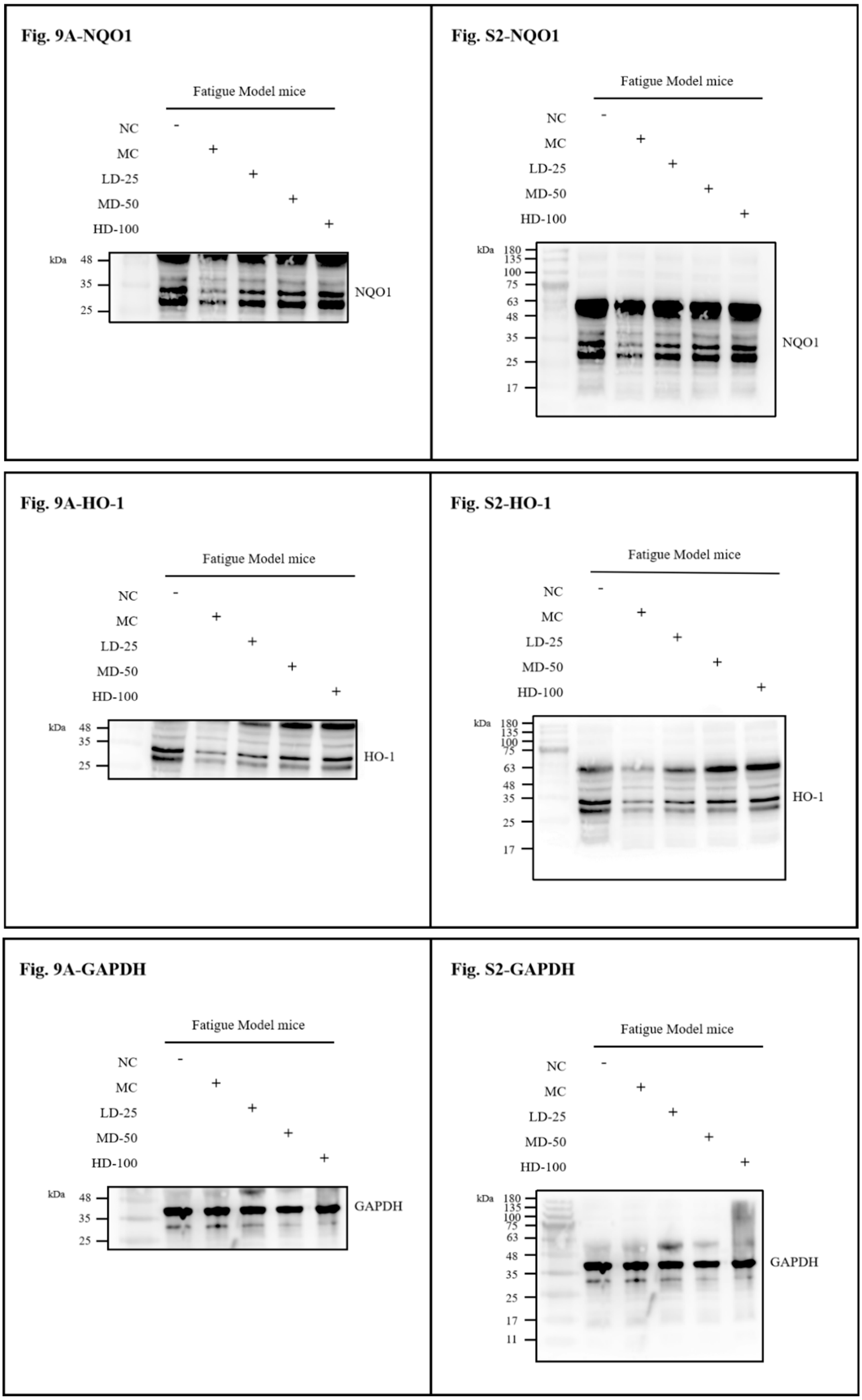

Fig. S2. Raw Western blots depicting the impact of PPPs on Keap1/Nrf2 pathway protein expression (Keap1, Nrf2, NQO1, HO-1) in fatigue model mice.

Fig. 10A-p-AMPK

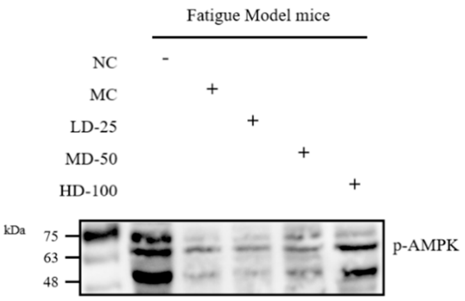

Fig. S3-p-AMPK

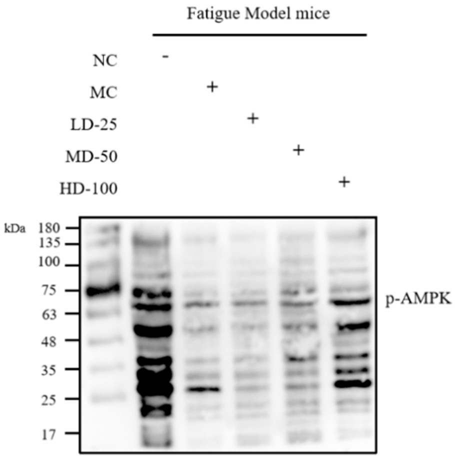

Fig. 10A-AMPK

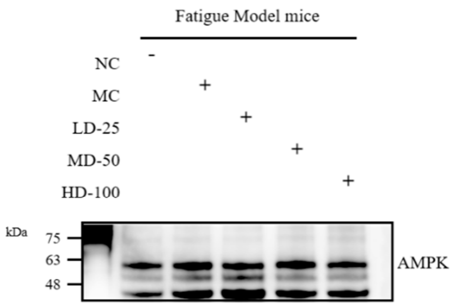

Fig. S3-AMPK

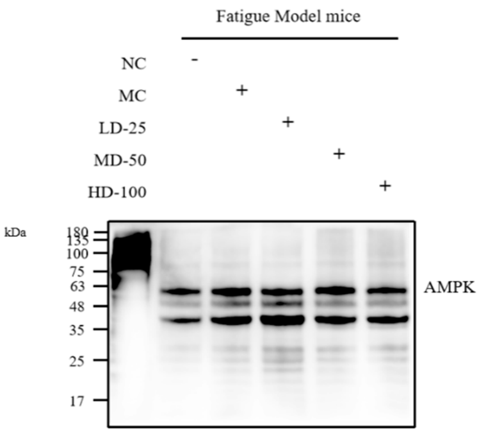

Fig. 10A-GAPDH

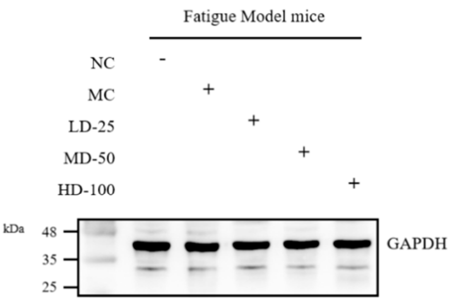

Fig. S3-GAPDH

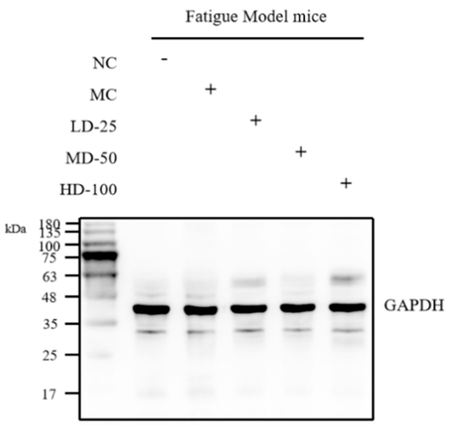

Fig. 10A-TFAM

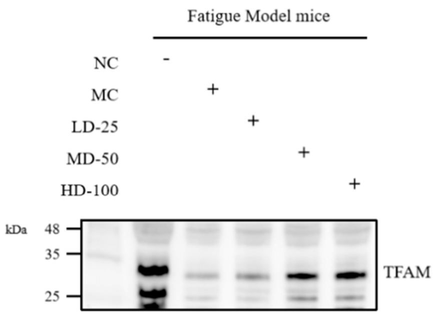

Fig. S3-TFAM

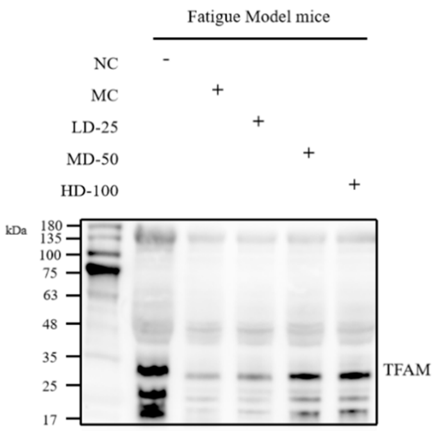

Fig. 10A-PGC1-α

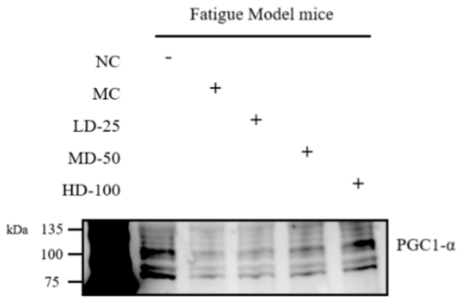

Fig. S3-PGC1-α

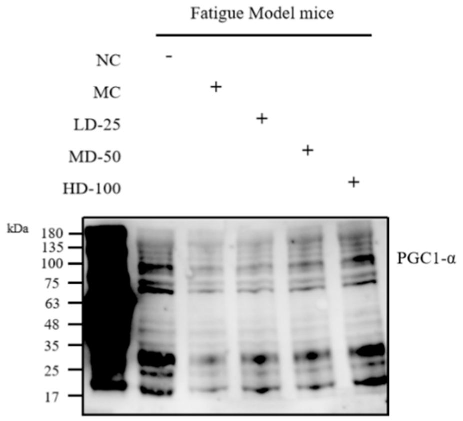

Fig. 10A-β-actin

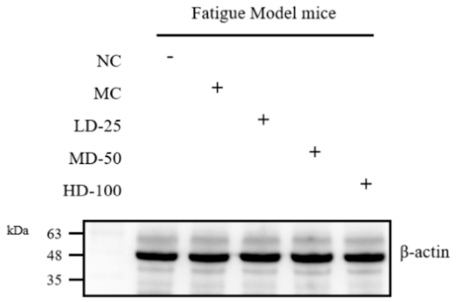

Fig. S3-β-actin

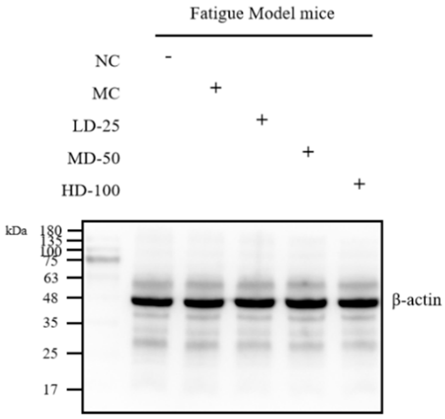

Fig. 10A-CPT-1

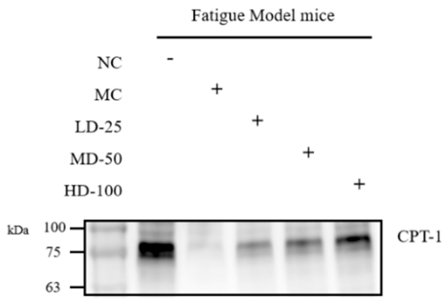

Fig. S3-CPT-1

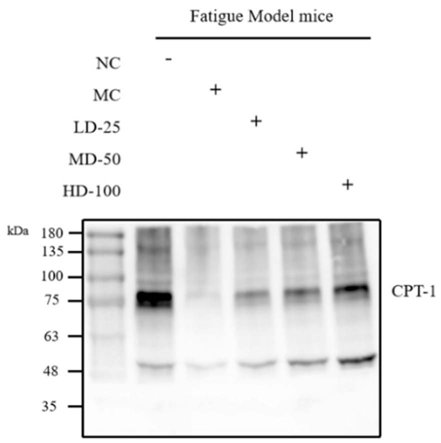

Fig. 10A-PPAR-α

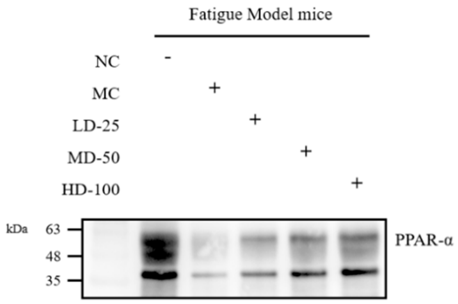

Fig. S3-PPAR-α

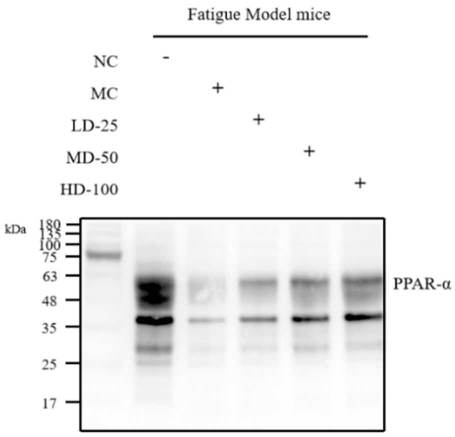

Fig. 10A-β-actin

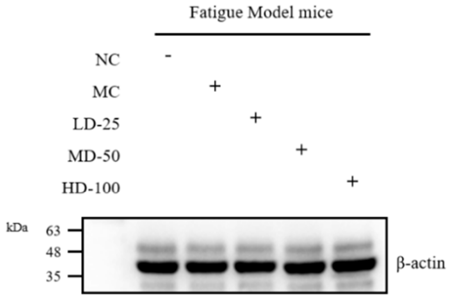

Fig. S3-β-actin

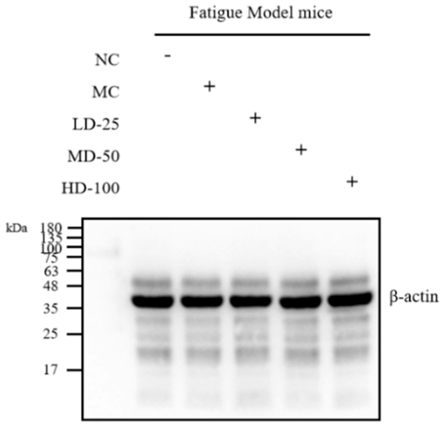

Fig. S3. Raw Western blots depicting the impact of PPPs on AMPK/PGC1-α/PPAR-α pathway protein expression in fatigue model mice.
